# Time collection and storage conditions of lipid profile

**DOI:** 10.1590/1414-431X20176955

**Published:** 2018-01-11

**Authors:** C.N. França, C.C. Mendes, C.E.S. Ferreira

**Affiliations:** 1 Pós Graduação em Ciências da Saúde, Universidade Santo Amaro, São Paulo, SP, Brasil; 2 Setor de Lípides, Aterosclerose e Biologia Vascular, Disciplina de Cardiologia, Universidade Federal de São Paulo, São Paulo, SP, Brasil; 3 Laboratório Central, Disciplina de Medicina Laboratorial, Universidade Federal de São Paulo, São Paulo, SP, Brasil; 4 Medicina Diagnóstica Ambulatorial, Hospital Israelita Albert Einstein, São Paulo, SP, Brasil

**Keywords:** Lipid profile, Storage, Temperature, Stability, Refrigerator, Room temperature

## Abstract

The stability of samples is crucial for getting reliable concentrations of many analytes, including lipid profile. Thus, the goal of this study was to analyze lipid profile under different storage and temperature conditions. This was a prospective study with 809 patients of both genders. Total cholesterol, triglycerides, high-density lipoprotein cholesterol, low density lipoprotein cholesterol and non-high-density lipoprotein were measured within 1 h from collection at room temperature, after 2-3 h of refrigeration (8°C) and after 4-5 h at room temperature. The processing time and storage conditions did not affect the analytes measured. These findings are important for multicenter studies, because of the difficulties related to centrifugation and freezing of samples immediately after collection.

## Introduction

Lipid profile assessment is an important tool to help in the diagnosis of cardiovascular diseases. Thus, the stability of samples is crucial for the analysis of total cholesterol (TC), low density lipoprotein cholesterol (LDL-C), high-density lipoprotein cholesterol (HDL-C), non-HDL-C, and triglycerides (TG). Stability is the capacity of keeping the concentrations of analytes minimally affected, within an acceptable variation, during a period of time (1).

Many studies have shown that plasma or serum should be quickly isolated from cells, within 2 h at maximum (2). Temperature and storage are two crucial points to be considered to guarantee representative samples, keeping their composition and integrity during the pre-analytical phase (3,4). Studies show that 75% of the errors concerning processing of samples occur in the pre-analytical phase (5,6).

The literature is poor concerning the stability in serum and plasma for biochemical markers, such as lipid profile components (7). Besides, the findings are controversial. Evans et al. (8) evaluated the effect of serum storage at −70°C on lipid profile and apolipoprotein concentrations. The authors showed that total cholesterol is stable for 6 months in that temperature, but there was an increase in triglyceride levels. On the other hand, in a similar study, Stokes et al. (9) did not find differences in lipid concentrations after 18 weeks of storage at −15°C.

There is no consensus concerning the maximum storage time and temperature for keeping the integrity of samples, beyond the differences in the precipitation methods. Also, plasma and serum might have different behaviors during longer storage, in lower temperatures (10). These issues need to be clarified, since multicenter studies frequently do not have the necessary conditions to centrifuge and freeze blood samples immediately after collection (11). In addition, errors concerning sample storage may cause an increase in costs and delays in the testing process (6).

Therefore, the aim of this study was to evaluate the effects of processing time and storage conditions for serum determination of CT, LDL-C, HDL-C, non-HDL, and TG.

## Material and Methods

This was a prospective study of 809 patients (62% female), age between 20-85 years (mean±SD: 63±8) at the Universidade Federal de São Paulo, SP, Brazil. As inclusion criteria, patients were seen at the ambulatories from this university; as exclusion criteria, individuals who refused to participate. This study was approved by the local Ethical Committee, the patient participation was voluntary upon signing the consent form, according to the Hensinki Declaration.

The samples were collected using traditional methods of antecubital venepuncture, under aseptic conditions, at a temperature of 23–24°C and a controlled humidity of 40-50%.

All quality controls were performed to ensure the accuracy of the analytical testing (internal and external controls). The internal control is routinely processed every 24 h on two levels (normal and pathological). The results are analyzed daily and periodically for the evaluation of the Levey Jennings graph. The laboratory's external quality control is performed for the lipid profile equipment every 3 months, with samples from Controllab® (Brazil). The laboratory where the study was developed is accredited by the PALC (Clinical Laboratory Accreditation Program) of the Brazilian Society of Clinical Pathology/Laboratory Medicine.

The samples were processed after 1 h of collection at room temperature, after 2-3 h of refrigeration (8°C) and after 4-5 h at room temperature.

Analytes were assayed on the Cobas C-501® equipment (Roche Diagnostics, Switzerland), using kits provided by the manufacturer.

Total cholesterol was evaluated by enzymatic colorimetric method. Cholesterol esters were cleaved through the action of cholesterol esterase producing free cholesterol and fatty acids. Cholesterol oxidase catalyzed the oxidation of cholesterol to cholest-4-en-3-one and hydrogen peroxide. In the presence of oxidase, the hydrogen peroxide formed affects the oxidative coupling of phenol and 4-aminoantipyrine, forming a quinone-imine red dye. The color intensity is directly proportional to cholesterol concentration, and the absorbance reading is 512 nm.

HDL-C was analyzed by homogeneous colorimetric enzymatic method. In the presence of magnesium ions, dextran sulfate selectively forms water soluble compounds with LDL-C, VLDL-C and chylomicrons, which are resistant to polyethylene glycol-modified enzymes. Under the influence of the cholesterol enzyme, the cholesterol esters are quantitatively decomposed into free cholesterol and fatty acids.

In the presence of peroxidation, the hydrogen peroxide generated reacts with 4-aminoantipyrine, forming a purple-bluish dye that is directly proportional to cholesterol concentration and is measured photometrically.

Triglycerides were measured by colorimetric enzymatic method, which utilizes the lipoprotein lipase for rapid and complete hydrolysis of triglycerides into glycerol followed by oxidation to dihydroxyacetone phosphate and hydrogen peroxide. The hydrogen peroxide reacts with 4-aminophenone and 4-chlorophenol under peroxidase catalytic action to form a red dye. The concentration of triglycerides is proportional to the intensity of the color generated and measured photometrically.

LDL-C was estimated by the Friedewald formula: LDL-C = total cholesterol – HDL-C – VLDL-C (Triglycerides/5).

Comparisons between groups were performed by ANOVA test. A P value of less than 0.05 was considered significant. All analyses were made using SPSS version 18.0 software (USA).

The study was conducted in accordance with the Helsinki Declaration for experiments involving humans.

## Results and Discussion

**Figure 1.**
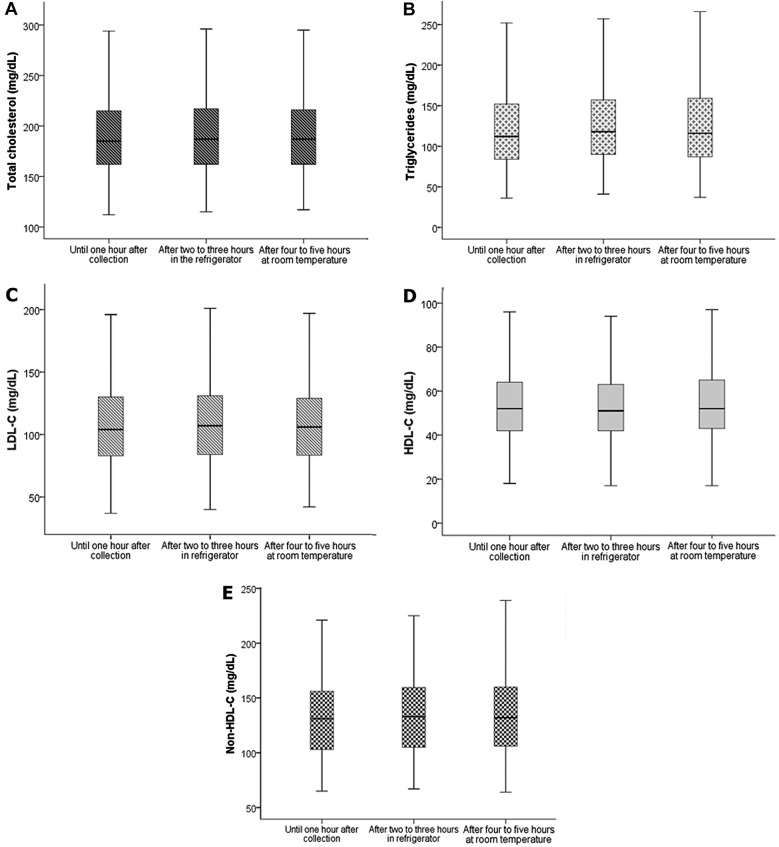
Total cholesterol (*A*), triglycerides (*B*), low-density lipoprotein cholesterol - LDL-C (*C*), high-density lipoprotein cholesterol (HDL-C; *D*) and non-HDL-C (*E*) were analyzed at three different conditions: within 1 h after collection, after 2 to 3 h in the refrigerator and after 4 to 5 h at room temperature. Data are reported as medians and interquartile ranges. There were no significant differences among groups at the conditions evaluated (ANOVA).

Figure 1 shows the results obtained. The processing time and storage conditions did not affect the measured analytes (P>0.05). In line with the results from Kuchmak et al. (12), we did not find changes concerning TC and TG in serum samples frozen at −20°C during 1, 4, and 34 weeks. Similar results were found by Ono (1981).

Heins et al. (13) evaluated the effects of temperature and storage time in many serum analytes, including TC, TG, and lipoproteins. There were no differences in TC, HDL-C and LDL-C after 7 days of storage in the refrigerator. However, at room temperature, TG and HDL-C concentrations increased (after 2 and 3 days, respectively) and LDL-C concentration decreased (after 2 days).

Another study conducted by Evans et al. (14) evaluated TC, TG, and lipoproteins. There were no differences in TC and TG after storage at 4°C for at least 10 days and at −20°C for at least 3 months; however, there were changes in lipid concentrations within lipoprotein fractions, mainly at 4°C, with increases in LDL and decreases in HDL. The authors suggested that a redistribution of lipids occurred between lipoprotein fractions. In our study, changes in lipoproteins were not found. These contradictory findings might be explained by the differences in the time of storage, since we processed the samples within 5 h maximum.

To the best of our knowledge, this is the first study to include non-HDL in the analysis of storage and temperature effects. Our study included 809 patients, whereas previous studies included a smaller number of participants (a maximum 106 individuals).

In conclusion, our study showed that storing serum samples for a few hours either at room temperature or in the refrigerator did not change the concentrations of total cholesterol, triglycerides, HDL-C, LDL-C, and non-HDL. Additional studies with whole blood and longer storage times are still necessary to optimize the inclusion of patients in large cohorts.

## References

[1] Associação Mercosul De Normatização (2009). Laboratório clínico - Pré-analítico. Parte 4 - Critérios de rejeição para amostras biológicas.

[2] Young DS, Bermes EW, Burtis CA, Ashwood ER (1999). Specimen collection and processing: sources of biological variation. Tietz textbook of clinical chemistry.

[3] Ono T (1981). Serum-constituents analyses: effect of duration and temperature of storage of clotted blood. Clin Chem.

[4] Stokol T, Nydam V (2005). Effect of anticoagulant and storage condition on bovine nonesterified fatty acid and b-hydroxybutyrate concentration in blood. J Dairy Sci.

[5] Bonini P, Plebani M, Ceriotti F, Rubboli F (2002). Errors in laboratory medicine. Clin Chem.

[6] Cuhadar S, Koseoglu M, Atay A, Dirican A (2013). The effects of storage time and freeze-thaw cycles on the stability of sérum samples. Biochem Med.

[7] Zivkovic AM, Wiest MM, Nguyen UT, Davis R, Watkins SM, German JB (2009). Effects of sample handling and storage on quantitative lipid analysis in human serum. Metabolomics.

[8] Evans K, Mitcheson J, Laker MF (1997). Effect of storage at -70°C on lipid, lipoprotein and apolipoprotein concentrations. Clin Chim Acta.

[9] Stokes YM, Salmond CE, Carpenter LM, Welby TJ (1986). Stability of total cholesterol, High-Density-Lipoprotein Cholesterol, and Triglycerides in frozen sera. Clin Chem.

[10] Ekbom T, Lindholm LH, Lanke J, Nilsson-Ehle P (1996). Decrease in high density lipoprotein cholesterol during prolonged storage. Scand J Clin Lab Invest.

[11] Ehsani A, Afshari A, Bahadori H, Mohri M, Seifi HA (2008). Serum constituents analyses in dairy cows: Effects of duration and temperature of the storage of clotted blood. Res Vet Sci.

[12] Kuchmak M, Taylor L, Olansky AS (1981). Low lipid level reference sera with human serum matrix. Clin Chim Acta.

[13] Heins M, Heil W, Withold W (1995). Storage of serum or whole blood samples? Effects of time and temperature on 22 serum analytes. Eur J Clin Chem Clin Biochem.

[14] Evans K, Mitcheson J, Laker MF (1995). Effect of storage at 4°C and −20°C Con lipid, lipoprotein, and apolipoprotein concentrations. Clin Chem.

